# Supplementary material for: A single vertebrate DNA virus protein disarms invertebrate immunity to RNA virus infection
Source: eLife. 2014 Jun 25;3:e02910. doi: 10.7554/eLife.02910 (PMC4112549; doi:10.7554/eLife.02910)
Supplement: Supplementary file 1. — L. dispar transcript sequences identified by mRNA-seq and primers used for dsRNA-mediated RNAi of L. dispar transcripts. DOI: http://dx.doi.org/10.7554/eLife.02910.025 [file elife02910s001.doc]

**Supplementary File 1- *L. dispar* transcripts Identified by mRNA-sequencing**

The sequences listed below were compiled by mRNA-seq analysis as described in Materials and Methods. In brackets next to each transcript name is the GI accession number and name of the top Blastx hit after translation of the transcript sequence. The species corresponding to the top Blastx hit is also indicated. Below each transcript name is the forward (PF) and reverse (PR) primers (5’-to-3’) used to generate dsRNA targeting each transcript.

**Relish** (gi|346987771| relish [Helicoverpa armigera])

PF: TAATACGACTCACTATAGGGATGCAACAAGACGCCATGAACGC

PR: TAATACGACTCACTATAGGGGTAGGCTGGGGTCCTAAATAC

TTTTATTGATATAATTAAAAAAAAAAAATTCGATAACGCGGTATCATTTTACGATGTGTA

ATGATGACCTTCACACAAATTTTATGTGCCTGAAGCTTTATATATCTATAGAAAGGGTTG

TAACGAAATACTGTGGTTGTTATTTGAATATAGCGTCGATTAATATATTTTTGGATATTT

GCAAGCCTAATCATAGACACTAATGCTCTCTTTATTTATATATTTCAATATAAAACATAA

TTTTGAAGGACTATTGGCAACAATGGGAAACATACTTTAAAAAAAAATCTAGTTGGACTT

CATTTTATGGTGTGTGCGCGAGAAATTTCTAGGGAAATGTTTAAAAAAGTACACTAATTT

TTACTAGTAAATTATTACCTATAATATTTACCATTAAATATATCTGAATATGAATATTGA

TCATCCTTTTCCACAGATAAGTAACCCTAAGGTAGTTTGTAAGCAATGACAAGGTAAAAT

TTGTTTCATTTCAAACTCAAAGTTTCTATTTATTTCAATGTTTTATTAACAATCATTTCG

TTTACTAATATATCGTGAAACGAAAACGTTCCTATTCATGACCATAGGATTTTTGTATTT

ATTTTTAATGCATAATAAATTATAAAGGACCATCCATAAAGTACGTTTAGATGGAGGGAG

GGGTCCTACGAAGCGTGATAACGGGTGAGAGGGGTTACTGTTCCAAATCTTACACGTCTA

AGTACTAAAATGCAATTTTAGAACTAGGTACCCTTCATTAGCATCAACTCGTTTCCAAAT

GCTATCACACGTGCCGCTTTACATAGTGATTTCATCATTAATGTTTTGTAAAATTGCAAA

ATATTTGACTTTTTTGACACTAGAGAGATGTCACTCAAATGTGGGTCACCCACTGTGACC

AAGACGAGAAACGGGTCAAAAATCACGAAATTATTGTGACGTACTTTATGAATGAACCCT

AATGTGAAATTTATGGGTGTTTGCATGATGAAGATGTATTTATAGCTGCAGTATAGATGC

CGTTCTTGCATACATTAATTATTTTATCCTATATTTCGCTGTTGCTTATAATTAACGCCA

ACTTCAATAATCAAATAAAAGATACTCTTCAAGTAAACTGTAATCTAACAATGTAATTAT

TTATACTGCTATTATCTTTATTTGATATTTTACGAATACCATATATCATACGATTAAAAT

ATCATATGTTATACGACTTTATAATTACAACGATAATATTGCTTGCTACGAATTGTGATT

ACTTTCTTACAATTTTATATATAAATTCCTTATATTATTAGCATTACATGTTTATATTAA

ATATACAAACTTCTGGAGTTCATTTTGAATACTTTATGTATGACAATATATACTTTCAAA

TATTTTAGAGAGTATTGGAAATAAAAATAATTGTATGGGATTATTAGTCTCAATTATCCC

AAACTTATTGATACTTAGTTATAACATTAGCATGATCATGGGATAATCTTCCTTGAACAT

TAATAAAACCCATACAATTATTCATATTCTATAAATACTTATATTGCATTAGAACATACA

ATCTGTGCTCTTCTCAATTTTATAATTGTATTAGTATTTATTGTAATGAACATTAAGTTG

AAAGTGTTACTCATAAAAACTTACTTTCTTCATCTATTGATTGCCTCCAGCATAAATTGT

GCAACGTTCCGCACTCTGTCTATTTTGGGCAGCATTCGATTTTATCATTCATTTCTCCTT

ATTTCAGTGGTCTATATTTCTACGTGAACTATACAAGAATTAAATTATTAAAAAATAAAA

ACAAGAATTAAATTGATGATTATTGTTAATTAAGGGCACAAGTTTTGCAGACATTAGATT

TATTTCATGTTTTAATGGCCCACTACATTCGTTTTATTTTTGTGTTTAAGATATACCTCC

TCAAAATCTTTGCCGATACTGTTACCTCTGGCAATGAATTTTTTTCTATACATAACTTTT

GTTTGAGCGTTGAACTCTATATCCTAATCAATAAAGTACAAAAGGGACGTTAACTTTGTG

TTTCCTCACAAATAATAAAACTCGCTTAATTTTTATAACCATGTTAGACTAATGAACGAA

ACATCAAAAAATAGTACTATTTTAAACAAGTTCCTAGCTTTGTGCGTAATAAACGATTTG

CTTAAACGACTGAAGAATTACAGACAACTTAGTGTTAACTACTAAGAACTAATAAAATAA

AAATTACATGAAATTGTTTTAATTTAATATAATGGTTGCCGGTTGCAATGTCGATTTGTA

ATTATTTCCGAACAATGTATAGCGCAATGTTTATACATATAAAATCATAAATAAATTGTA

GTTTTATTTTTCTTAAGAACTTTAGTTGTATTTATCTGCATACAATACTGAATCACTACA

ATTTTCTTGTATTCGAACCGATGTTCGACATTAATATACTCTTTAAAGTTAAAATACTTA

ATTTTACTTTATATTTTATTGCGGATGCCATTCCAAAAACCTTTGTAAAAGGTACAATTC

TTGTCAGTAGCTATACGACTTTGTTTTTATTATCGAACAAAATGTTATTATTGTCGTTCA

TTTTAATTATAATTTCAATTTGGATAATAAAAATAAGTTAAATGACTTGTAGTATTATTT

TCTTGTTTCCACACATACGATTTACGTGTTTTTCAGAAGGAAATTTACATGTGGCCGAGA

ATTCAACTCGGTTGGAATAATCGTTGTGATGACAAGGACCACTTCCTCTTCGTGAAACCC

GGGCTGGAGAATTAGAAAGATCGATAGAAGCACTCTCCTGCAAAACTTATAATACAAATA

CGTGGTTATATATACGTTGAAGGATCTAATTAAAATATATTTGGTACATTAAATACGATC

TAAACAATGCTTTCCCAGAGCAATACTTAAAAAATTCAAAGATCATTTCAAATAGTAAAT

TTTTCCACAGACGGCAATACGATATATTTTTTTTTTTAATAATCTTCATTTACAAACAGA

CAGACCGCGTGTTTGTTAACCCTGGCTACGTAAAACGAAGCAAGTTAATTTATTATTGAC

AGCTGATTAAGATAGTAGGTGTATTTATCAGTTCGAAGATAAACCTATGGGTCAACTAAG

GGAACAGTTCTACAGCAGTTTGAGATCAGTTAACGCGACTCGAAAATAATTAAAAAAGTA

AAATGAAAGCTGTCGAGACAAGAAGATTTTTGGTACGTATAAATATACTTTTAAAGTAGA

TAATAATTATTTTAATGAAAATGTAGGCATCTAGATGGTGTTAAAATAAAAAAAACATAA

GTAGTTTTATTAGATGTACTAAATTGTAATTAAATTATTGCTTATGTGTACGTAGTAGAT

ATGTATTTTATATATTTTTTTATCTAGATGCCTACTAAATAAAATAATGAAATGTATAAT

TTTAAGACTAAATAATATTCTCTTAAGTGGAATTTAACTTATTGACATAGGTAACTCGCA

TCTTTAACGAGTCATGCTACCTACCAATTTAATGGGACGCCTTCTCTGTTTGTGAATAAT

AAAATTATTGTAAATTCTCCGTGGAGTAGTCTTTATTACACCCTTTCTTTATTTATTCAA

TAAGTTCTCATAGGTTATTCGCGTTCAGCTGGTTAGCGCCGGTATTGTAAACGTGATGAA

TCGTATTAAAATGTAAATAAATTAAACGAATCGCTTACCTGGGAATAGAATATAATAATT

ACCGCTTCTGTAATACAAATAAAGGTATACTATGTTACCTACCTATATGTAGATTAACTG

CTTATTTTATAAAAGCAGATACATCGGAGCTAGTATACTTTTTTGGGTGTATTTTATTAG

GCAAACGTAGACGTTAAAATGGCTACCTTGTACGTGTCTATGACATTGAATTGACCTTTG

AAAGTCCTTGCATTACACAGCTGCTGTCAAACTGCAAAGAACGGTGCGTGTGAGAGTGGT

TTAAAGATTTAATTCAGTACAAATATAAATATTAAATAATATTTGATCACGGCTTAGACT

GGTTCATCTGAGAAGGCTGCTATGCAACAAGACGCCATGAACGCTGGATCCCATCTACAA

CATATCTGTGGCCTGGATATCGACTCCAAGGCATCTTACGTCCGCCTGTCCGGCATCATC

TGTACCATTGAAAGCGCATCCACTAATGATTTGAAGATTTGTCGTATGAGCCGATTTTCT

GGCGACGCGTCTGGTGGTGAAGATGTCTTCATATTGGTGGAGAAGGTCAATAAAAAGAAT

ATTATGGTACGGTTTTTTGAACTGGACCATAACGGTGAGAGGATTTGGGAGAGTGTCGGA

CAGTTCATGCAGACTGACGTACATCATCAGTATGCTATAGTATTTAGGACCCCAGCCTAC

AAAGACCGTAAGACTCCAGTGGATGTGCAAGTATACATAGAATTGGTACGACCTTCGGAC

GGGCGGACCAGTGAACCGAAAACCTTCAAATATAGAGCTGATCCGATGCATATGCAAATT

AAGAAGAGAAAAGCCAACTCATCCAATTGCTCCATTGGAAGTTCTTCCAATGGATCTTTA

AACAGCACTTCTGATATCCCAGTTACGGTTATCAAACATCAAGATGAAATGATGGCAAGT

CCCATACCAACTGTACCTTCTCCATATTCACATTATGCGGGTAGTCAGATGATGTACCAA

ACATCTCCAAGCCAATGCGATCTTGCGAGTGCACTGGCGAGCTCGGACTCTAACCAGTCA

CCTGTACCCAGCCCTATGTGGGGTCAGACACCTTACACCCTACCACCTACCGAACCCAGC

TTGACGGACCTCCATCTCAACTCTGCTGATCTCGACATCTTGCAGAACACAGAGCCGGAG

AATAAAATTTTATCAGAGGGCATGTCACAATTCTTTAGCGTGTACCTAAAAAGTTACAAC

GAAGAATATCCAGGCGATAAAGCTCTAGAAAGTCTGGACTTTAGCTCAGCGGCAAATATA

GTCGGGGATTCGGGTCGGCCATCTCAAGTTAAAGTCAAGGATGAAGTTGAAGTGCATTTC

AGAGGCTCGTCGAAAATTCCCGAGTCAAGTCACGCCCCAAATGATGCCGATAAACAAACA

AACGCGAAAAATCCAGCAATGTATAACGCTTTCTATAAAACTGAGGATGGCGCAGAAGTG

AAAAAGCTCATCAAGGACCTGTGCGATATGATAAGAGACAAAAAAGGTTATAAGAAACAA

GAGGTCAGAACGCGTTTAGAGAAATTGTTCAGCATTCGTTTGTCTAACGGAGATACATTT

TTACACATGACTTTATATTCCAATCTGACTACTTTCGAGTATATTGTGAAGATCATTCAC

AACGTGAAGACGACTCATCTACTGGATTATACCAACAAAAGTCAACAGACACCGTTGCAT

CTTGCTATACTTAACGACATTCCTAGGATAGTGACATTACTTGTCTCTAAAGGCGCTAAT

CCCATGATGAAGGATGTTGAAGATCTTAACGCGATTCACTACGCCGTCAAGTATAACTCG

TGTTTGGAGCCGTTGTTGGATGCGATCAAGAAATATTGTGTGCCTTGCGACCTGAACGAT

ACCAACAATGAGAAGCAGTCCGCCTTGCATCTGGCCGTTGTATTGGGTTCACAGCGTAGT

GCATCCACGCTGCTTAAATACGGTGCAAGTTATAGCGTCCGTGACTCTCAAGGACGTACA

CCGCTACACATTGCTGCCTATGACGACCGGCTTGCCGTCATAAAGACCCTACTGGACCAT

ATACCACTGAGCGAAGTGGACGTTGTCGACGACGCTGGCAACACCGCGTTGCAGATTGTT

TGCGGCAGTCAAACGATTAGAGAGAATACAGTCGCTATTGCTAAACTTTTGCTGGAAAAT

AAGGCTAATCCTTTGAAGGATGAGGAGGCTACGGAGTCAGCGTGGCGTTTGGTCAGACAT

AAGCCAGAACTGAAGTCATTACTCGAAGAGTATGTCGACTCCAGTATAATGGATGAAGAC

GACATCAAGTCTGAACCGGATGATGATTTTGAGTCTGCTGATGAAGGGGAGCTCCAGGAG

ATGGGTATACATGACTTAAGCCTGTACAGTCGTGAAGTATCAGTGTTACTCGATACAACT

GGCGCTTGGCGTTCCCTCGCCAAGCGATTGAGGCTGGATGCTTTATTGGAGTGGTACGCC

ACACAACCCAGTCCAACGCTCACACTACTCAACCATCTCAAGGATTCAAGAGATGATATC

TCATCGAAATCATTGGTGCTGATACTAGAAGATTTGGGTCAGACGGAAGCGGCCAAAATC

ATTAGACAAATCATCGAATGACATCACAAAGAAAACTGCCAATTAATTATAAGCTAGCAT

TTATCAAATCAAATGTTATTGGATTCAAAATCTAAACGATAAATATTTTTTTATAATGTT

AATCTGTGACCAGTGAACCTATCGGATGATAATTTAAAAGCTAATTAATATTCTTTAATT

CCTACCATAATTTCTATGAAAGTTTTTTTTTATGCTGTTAAACTTGTTTCTTGCCTACTA

CCATAAATGAGTCGGCTTTTCGCAATATATCAACGTTTTGCTTAGGTTGGTGCCAGAGTT

AGCAGATAGTCTGAAGTGAAAACAGTCTCCACCTCAGGTCCCGTCCTCATTTCATCCCTG

TTACGATAGATAGGAATCCTAACCCTAGTATTGCTCAAGCTAATTGAAAAATGACATTTT

GTAACATTAAGGAATAATACAACACTATGTTACTATTACTGATTTTACAATCATAAAATG

AATACACACACAACATTGAAATATTTGTGGATCACACAAAACTTTGCGCCGCGCTCAAAT

ATTTGTGAACCACTCAAATATTGGTGGCACCCCAAATATTTGTGGGCCGCCCAAATCTTT

GTGAATAACTCAAATATTTTTTATTCGAAATTGGATTTATTTAATTCGCTAAATGGGCCA

CACTATTTAATCATGATGCGACATTAAAAGTATAATTAATTTTTATTAGTCGCGAGGTGA

TAGGATTTAAGGTGGGTTGTATGAACTTTGGTCTACACTCTATAATCGAGTCTATTAAGC

ATATTTCTTACCATCAGATAGGTTAGCTGGCTCATTTCTTACAATAAACATTAGAAACAT

ATTTTTATAGAATTGACCATAAAATTAATATCAAATATGTAAGCATTTTTATGACAAAGA

AGACCTTCACAAATTTTTAATTTTGAACCATATGGCTTAAAATTAATAAATAAAATATAT

GTTTATATAATTTTGTTTAATCATGACACCTGTTTACGCGGGTACATTTGAAATAAAATC

ACTTTTGTTTTTAATTTCGTCGCGTTTCAATTTTGTTTTTACTATTCTGATGACCAAACA

ACGGTTTTCTATATCTTTTTCATAATGGACGTGTTCCAATTTAGTGGTAGCAAGTTAAGA

TTAAGTCTAATTTACCTACTATAAATCTACTGTGACATCCTGTTCAGACGTGTTTGTATA

GGATTTTATTTCGAGTTTTTCTTGTATAGATGTTACCTGCAATATGTAGAGTCGTCTTTT

ACATTCAAATCATTTATTATGTATAAACCCCGCGGGGGCACTTACATATTGTCA

**Effete** (gi|357604946| effete [Danaus plexippus])

PF: TAATACGACTCACTATAGGGATGGCGTTAAAACGAATTAATAG

PR: TAATACGACTCACTATAGGGCAATTGACTCATCACATGGC

ATGGCGTTAAAACGAATTAATAGGGAATTACAAGATCTGGGCAGAGATCCACCAGCACAATGTTCTGCAGGCCCACACGGTGAAGATCTATTCCATTGGCAAGCCACAATTATGGGCCCAGTTGACAGTCCTTATCAAGGAGGAGTATTTTTCTTGACCATACATTTTCCGACAGATTATCCATTCAAACCACCAAAAGTTGCATTCACAACACGTATTTACCATCCCAATATAAACAGTAATGGTTCCATTTGTCTTGACATTCTGCGCTCACAATGGTCTCCAGCACTAACCATATCCAAAGTGTTGCTCTCAATCTGCTCACTCTTATGCGATCCAAACCCAGATGACCCATTGGTGCCAGAAATTGCTAGGATCTACAAAACAGACAGAGAAAAGTACAATGAATTAGCCCGTGAGTGGACGAGGAAGTATGCCATG

**AGO1** (gi|357618035| argonaute 1 [Danaus plexippus])

PF: TAATACGACTCACTATAGGGTCGATATCGTGTCCCTACTTATG

PR: TAATACGACTCACTATAGGGTGACAATGCTTGACTAATGAGC

TCGATATCGTGTCCCTACTTATGAATATGGCATTGTCACTCGTTTAGTTTAACACTTCCA

AGTAAACATTTTAAGTAAAATACAAAAAGGACGAAATGATTATAATTGATTTAACTTTCC

AGGGCCAGATCTGTATTATAAAAAGGTGGGAAAACTGTGAATTAAACTCGAGACAAGATT

TTTTTCTAGGGAGGACTTCAGAATGGTGCTTCGACTATTACATAGCCAGAAAAGGACAAT

GCTTGCCAACAAGCACTTAAAGAAATAAAAGGGACAGTGAAACACATATCTCTAAATGAG

ATATTCATGGTCGACATCACCTCTGAGGCGTGCGATCAGGAAATAGGCGGAGTTCCATCT

CACAAACAAGATAGATCGTAGAGATCATAGTCTTTATTGCTCATTAGTCAAGCATTGTCA

CGCAACATAAAAATATTATGTATGTCTTAGTAGTCACCTCAAGTTTATATTGTTTCATAA

ACGAAGTATCCAGAGAACGCTCCATAAAAAATAGCATACCCAGCATCACCAATAATTGCC

TCATAGCAAATCACGGCTATTAGATATCCGAATTACGTCGATAACTGCGCACACGGACAG

AAACAAAAGCGGAATCCGCTGATGTAGGATTAAGATCCGCAGATGTGTTGCGCATGTTAC

CTGTCGTGGAGGCAAATCAGTAGCTGCTGTAGACAGCTGCCGGCTGGTATGGCGGTTAGG

GGTGGGTTAGGCGAAGTACATGACCTTTTTAGTAACGGCGTGCACGGTGATCGCGCGCGC

CATTGCGATGGGCGTGCGGTCTTCGCTGCAGGCGGACTGGTGGCTACCCTCGCCTGAGTC

GTGCTCCTTCTCGACGAGGTGGTAGCGCGCGCGGAATGCCACCAGATGTGCGTAGTAGGC

CGGCGCGGGAATCGACACTGAGCGCGTACAACGCACGTAAGTGTGACACAGCTGGTATGT

GAGGCACTGCAGCTCGTCCGAACCGAAGTGGTTGTCGTCCCAGAGTACGTGGTAATGGGA

CGGTCGCGACGTGCCCTGAATTCCTTGATGACTGCACAAATAGAAGTCAAACTCGGTTGG

ATGTGTAATACCCAGGTCGACCGTGGTGCCGGCGGGGATGTTGCCCGATTTGCCCGATTG

CTCCTTTTTATCTGCACAAAATAGCCTCGTGTGATGACGTTTCTGTACCACAATGAATGT

GATACCCGGTTTGTACTCAGCTTCCAACTTAATACAAGCTTCTCTAACAGCCGTGAGTTC

ATGTTGCAACACGTGTAAAAACTGTCCCTCGGAAATGCCGTCACGGTACATGATAATGCG

GTGTGGCTTGAATCCGCCTGTGCTCTTGTAAAACATTATTAATAGCTCTTGCACCATACT

GCTCATCTCATGAACGATTTCTTGTCTGTGCTGTTGCACGCGTACAGTGGCAGCATAGCG

TGATGGATGTGCGTCCATTGAACCAACAACGGCTGCGATGGACGGCTTCTTATTGTCACC

CGCAGGCGGATGTGTCACGTCCACGCCCAAGAATATCACCGGTTCATTGAAGACCTTTGG

TCGTAGCGACGGCACCAAAATGGAATTAATACCGCCAAGTTTCACATTGATCTTAAGACA

CAGGTTACTTAAAGTTTGTGGTGATGTCTTGTTAACATTCTTTGCTTGCACACACTGCGT

TGCCATGCCCAGTACTGTATCTCCGACTCGCTTTACTTCCGCATAAACAGGAGTCTTGCC

TGGTAGAACAACAACTACTAATTGAAGCTGGACGAAAGTGCTTTTGAGATATTTGAACAT

TGGCTCCACTTGATCCGGTCCAGTTGCATACTTGCAGAAACAAGGCTGACCTATGATAGG

CATGCCTGCGTCATTTGATATCTTCTGTAATTGTTGAGTAAAGTTCTTGAGGGCGTCTTC

TCTAACTGTTCTCTGTGGGGCGAAGCAAGCGATGGCCCATACTCGAATCTCGACTCCCAT

GAAGAATTGCTTGCCTCTCATATCCCAAACGCCTTGGTTAGGCAACGCTTGACCACCGAG

AGAAGAGACTCGCCCACCATACTGCAATTTAGGTGGCGGCAGCACTCGACCTCGGACTTC

CATCATGTTGTTGGAAATCGTCAAACCAAACTCCTTGACGTACAGGTCCGTGTTGAAATT

CGCTCGACGAACCAAATTGTTTATCTCTCTTTCTCTGTCAGGGGCAGAACGTGCTGTTGC

CTTAATCATAGTTGATGTTTGCATGTCAGTTAATTTTTTAATACATCTCTGTCCTGGGAC

AATATTACACACTTCTAGAGGCAAATATGTATGTTTGTGTTCTTGTCCTACTTGTAGACA

TGGTAAATGTGGATACCGTAACTTCATCTTATATTTGTCCATAAAATATTTTGCTACAGT

ACACTCAACTGTTTGACCATTTTCTAGTTGAAGAGGAAATGATTGCATTTGCGAAGGTCG

ACGTGTTACATTACAAACTCTGTATTTCCGCTTCATTGTACCACAGTGAGTAATTTCTAT

CTTGAGTCCTTTAATCTCCTTAGTGAATTTTACTCTTTGAGAGTCCGTTAAAGGCTTTCT

TTGATCATTTATGTCTCTTATGTCTAATACCTCACACATAAATTCTATAACCGGTTGTGC

TTTGTAAAAGGCTGTTGCAGAAACATCAATATTCAGCATCATTTTCCATTGACTGGGTCT

TACAGATTGATGAAAACCAAACCAAACTTCTCTACCTCCACCGAGGGGATGATAATACCC

TTCTGGAGATGAGAAAAATGACCGTCCAACAGGAGTATACATCATAGAAGGTAGATGCCT

CATAACTACATCTAAAGCTAAGATTGCATCGTAAGGAATTTGTCTTGTTCTTCCTTCTAG

AGCCTCTTCTAGTGCAAATAATGACACTTGTGCTACCCACTTTATGCTGACACGAAACAC

TCTGTCTTTGCCTTCTCCAGGTAATATAACCTCCAATTCAACTTTATC

**AGO2** (gi|166706854| argonaute 2 [Bombyx mori])

PF: TAATACGACTCACTATAGGGTCAATACTTCGGTTGAGATCAAC

PR: TAATACGACTCACTATAGGGCGCTGTTTCTCTATAGGCATG

ATCGATACGTACGTTTCAAATTTCAATCATCTGTGTTTATAAATAAGTAAGCTACCTGTA

GCCATTTAACAAGAAGAGGTATATGCGATCAGAGTACTAGTTAGTACATACGTATAAATT

ATAAAATAAAGTGATATATTCTAGTGATTCAAGTGATTGTATTGCTCTTGAGATTGCCTT

ATTGTTTTACAGAACCAATCATCAAAGATTAAATAATGCCTAAGCCTGGTAAAAAGAAAG

GTCAGAAACAGGAGGCCCCAAAAGAGGAGCCTCCAAAACAGGAGCCGTCAAAAGTTGAAG

AGGAAGAAGATGTTGGGCTTGGTCTTGGCGGTGCGTCAAAACGCAAGCAAAAGAAGGAAA

AAAGCCAGCAGATGTATGAGGCTGCAAGGCAAGCATCTACATCTCAGCAAGCTTCAACTT

CAAGAGAGGCATCTCAGACTCCTAAACCTGATCACGAAAGTAAAAAGGCAGAGGATCCAA

AACAGATACCAGGACAAATTGCCGAAAAGGCCGAAGATGAAGATGTTGGTCTAGGTCTTG

GTGTAGGATCAAAGCGCAAACAAAAGAAGGGAAAGAGCCAGCAGGTGTCATCTGATCAAG

CTTCATCATCAAGGGAACAATCTCAGGCGCCCGAAGAACCTCCTAAACCTATAGTTGAAG

AAATTACCTCTAAAGTTGAAGAAGTTGCCGAAGACTTGGATGGTTTAGGATTAGGTGGTG

CAAAAAGAAAACGACCTAAAAAGAAAAAAGCTGATGCCGCACCAGCAACCATGGTTGCTG

CAGCTTCTGCAGGCGAGCCTGCTGCTGCTCTTGAACCTAAAGCGACGCAATCTTACCAAC

AAATCCCATTTACTCCGCATCCACCACCTGGTTTTGCACCTCCTAGTGCGTCAAGTGCTC

TGCCAGCACATTCCCAGGGCTGGGGTAGAGGCAAAGGAAGAGGATACCCGGGGAGCGCGC

CAACCGTAAACCTACCATCAGTAGGAGCGCCTTCAGCCGTACCACCATCCGCAATGTCTT

CTCAAACTCCTCAGACTCCTTACCAACCGCTTCCCCAACAACAAACGCAAACTTACCAAG

GACCTCCTCAACAACAACAACAAATATCTCGACCACAAACAAGCCAACCACAAATACCAA

AGGGTCAATTTGCGCCTGCTTCTAACATTCTATGTCGATATAAAATACCTGCTAAAGTTC

CAGGTGGACATGTTAATGGTAGACGAATTATCGTAGTAACAAATTATTTGGAAATGAACT

TTAAACCACTGTCTATATCAAGATATGATGTGTCGTTTACTCCTGATAGGCCAAAGAAAA

TGCTAACACAAGTATTCAAATTAGTTAAACGTGAATACTTCCCTAATGAAATAACAGCCT

TCGATCAAGTGAAGAATTGCTACTCATTAAACCCATTGCCGAATTTTACACAAGGACGTC

TCAATACTTCGGTTGAGATCAACGATAGCAATGGAAAGTTGATGAAATTCGAAGTATCAA

TAAAAGCAACAGGTGTAGTGGAGTTGGGTAAGATTAAACGACATATGGCTGAACGCGGAA

CATCCTTGATGCAACCTACTGAAGAGATACAATGTATTGATGTCATATTACGTCAGGGAA

CGTTGGAGACTTATGTCAAGGCCGGTCGTCAGTTTTTCAAGCGTCCTACGAACCCTGTAG

ATTTAGGCCACGGTTTTGAAATGTGGACCGGACTTTTCCAATCTGCTATATTCACTAATA

GGGCTTTCATAAATATTGATGTTGCCCATAAAGGTTTTCCTAAGCAACAATCTATGATTG

ATGCACTAAAAGATTTTAATCTTCGAGCTGACATGCCTATAGAGAAACAGCGCAGTTATG

AAATCGAAAACTACTTGCAATTCATCAAGGGTCTAAAGGTCGTAGCACATTTAGTCGGGA

ACGCTCCATCTTCGGGACAAAAGCGCGAATTCATTTGTAACGGATTAGTGGATCCACCAG

GGAAACAAAAGTTCATACTAACCGATTCTGCTGGCAAGCAGCGCGAAATAACTGTTGCAG

ATTACTTCGCAAGGGAAAAACAGTATCGCCTTAAATTTCCAAACCTGAACTGCGTGTGGG

TGGGTCCCAAGGATAAGAACATTTACTTCCCAATGGAACTGTTAGAAGTAGCTTATGGAC

AGCCACTGGCCAGACAGTTGGACGATACGCAAATTTCGAAAATGGTGAAAGAAGCCGCGA

CTCCTCCGGACAGACGTTTACAGAAGATAATCGAAGTGATCTCCAGTATGAACTATTCGA

GAAATGCAGACTTCAAAAAGTTTGGATTGGAGATTTCAGATAAATTCTTTGAAGTTAATG

CAAAAATTCTGGAGCCACCTTCACTGGATATTGGTCGAGGAAGCGTTATTCCCAGAAATG

GCGTGTGGCAAGCCAACTCTCTTTTGAAAGCGCAAGCTCTTACCTCATGGGGTTTCATTG

CTATCGAAACGCAGCCCAATACAGATTTCAACAGTGTTGTTCACATGATTATGAGCACTG

CAAAACAAATTGGTATGCAAGTTGCGGAACCTAAATTAAGACGTTATGATGTTACTTTAA

CCTCTTTAAACAGCGTATTGCGTGGTGCCTTGGACAACGGCATCAATTTGGTCTTTGTAG

TTGTGTCTATGAGAGGGCGCGATCACTATCATAAGGTGAAACAAATGGCGGAACGAGAGG

TGGGAATGCTTACGCAATGTATTAAAGAGGCTACCGCACGTCGTATGAATCCTATGACAG

CGAGAAATATTCTGCTTAAGGTGAATTCCAAACTCATGGGTATCAATCAAGCATTGGGTG

ACAAGACCCTACCCCGTTGTTTAAGAGACGGAGGAGTGATGGTAGTTGGCGCCGATGTTA

CTCATCCTTCTCCCGATCAGGCTAATATACCTAGTATAGCAGCAGTAACTGCTTCAATCG

ATCCAAAGTGTTTCTCTTACAACATCGAGTTGAGTATTCAGACACCAAAGAAAGAGATCA

TTGTTGAATTCGAAGCTATGATTCTTGATCATTTGAGAGTGTATAGAGATCGGAATGGTC

AGTTGCCGAGAAAGATATTTGTATTTCGTGACGGTGTATCTGAAGGACAATTTGCTCAGG

TCATGAATAGCGAGCTGACTGCTGTACACACTGCGTATCAACGTATGGCAGGTGCTGGAA

AGAAGCCTGAAATTCTATTTTTACTTGTACAAAAGAGACATCATACTAGATTCTTTAATA

GAGGTGATTACTGCAAATACAACGTTGAACCGGGCACCGTCGTCGATACTGAAATTGTGC

ACCCAGCAGAATTAGACTTTTACCTGGTATCTCATCAAGCTATAAAAGGGCCCGGCCGAC

TCGCTACCACGCAGTGTGCAACGACGGTCGTATCCCTGACGACGAGGTTGAACAGCTGAC

ATACTACTTATGTCATTTGTATTCACGCTGCATGCGCTCCGTGTCTTATCCAGCTCCGAC

TTATTACGCGCATCTAGCATGCCTCCGAGCCAAATCGCTTACTTACGGTCAGAAGTTTAA

CAACAAGGATTTGGAGACTAGACCACAACGTCTACACGTACTTGACAAGATGTTACAGAC

CAGCCGCATGTTCTATGTTTAATACCCCGATATGTATAATGTAATTTCGCGCGAATTTAA

GCAAGTACTATTAAAAAAAAAATGAAAAATCAGATATAAGTATAATTCTGTGTTTGTTAA

ACTGTATATTTGTAGGGTACCTTGCGGAAAGTGTTAATGGTTAAATTATTATTCTATATA

ATATACTAATTTTCGATCTCTGTGGGTTGTATGAACTAGAGTGCAGTAGTTCTCTGATAG

GTATCTAGGATGAATTCTCATTGCTGGGCAGGCCACGGACTTCAAGTTTTCGATATCAGT

GGTCGGCAAATGGCAAAGCGTAGGAAGTACGATGGGTTATGCTTCGCGGCGCCCCTTCGT

CACCACCCCGGACGGAAGGGACGGCGCCCGTCTAGACTGTAGCCCACCCAAAAAAAAGAA

ACGATGAATTCTCATTAAATTGAATTGCATAATTTAAATTGAATTGTGTAGAATTGATTT

GTGTGCTTGTATTTTGTGATTGTTGCATAAATTTAAATCAAATTTAATTAATAAATTCTC

TTTTAAAAGCAAGTTATGATTTCTATTTACAAAATATTGTACTTGTTAATTTCGCGTTAT

TTTAATATTCAGGTTAATTTTTTCTTATATAAAATTTAAAAATAAACTATTAATTTTCAT

TTCGTTCTCAGAGTATGATGATTTCTGAAAATCGAATAAATATGTATCTTAAAAAAATAA

TAAAAATACGTCCTTGCTTTTGTGTCAAGGTAAAAAATAAATTATATTTATTGATGTTTT

CCTATATTAAATATATAAGTCAATAAAATCGGTTTAAATTATGTAAGAAATATTTGAAGT

AACTTTTATTTCATCCAATTATTTGGTTTAATTGTGCCAATCTACGGATGCGTTATCGGC

AGCGGAACGTAAATTGAAGCGTGAGGTTTGCCCGACTAGTCGTGGTTGATCTTATAAAAT

GTGTGATTTATATATTTTTCTTTATACCTTCCATTTTGTATTTATGGAAGTTAAATAATT

TGCCTTCTTTGTAAGTTTCATATAGTAAAGTACTACAGAGTGAAGTGTGTCCACTGCGCT

AAGATCATCATAATCACAGCTTATATACGTACCATTGCTGGGCTCGAGTGTCCTCCGTAA

TAGCACGCTGGTCAAATGGGATGGTATAGGATTTTTGAGTTTTCCTTCTCTTCCACAGAC

CTATTCTTACTCCCGTCACCACAAGGAAATGATGCCAAGATATCATTATCATTAATTGCT

GTGCTACTTTTTTGTGATTTCAATTAACCGCAAATGGTTATATGACAACTTTACCCGGAC

TGTTGAGTGAGCGCCGAGGGCTACCAACTCGATGATGTACCATCCTCAACTATTTCAATT

CATAATATTTGCATATTTTTTTAATGCACAATTTAAGGTTGTCTACCTTATTGCATTAAT

GTTTAACGTTTAATTGTATGTGCTCTCATGGTATATTGATTCACTGTAGAATTTATATAA

TGATAGTAATCATTTTGACATGATGTTAATAAACGTAACGCTTAAAATGTACCACAGAAA

TATTAATACTTTAGAAAGGCCCTTGCTCATACTAGCACCAGTGTCGTCGCATTTCTTCCT

CTAGATAATATTAACAGATACGCAAGGTCTATACTTATAACAATTTCACACACTAACATA

ACACATACACACATCAATCTCGAAGCTGCAGTTTGTTAACAAACTATAATTAACTTCTGT

AGCCAACGGACATTCGCACACATGAAAAAAGCACCTACAAGTTAGTGCTACCTGGGTGTT

ATCGCGGTGCTCGGTGATATCGCACTGAACACCGTACATTGCGGGGTTGTTCAAATAATA

AAAGATGACATCAATGTATGGTAGACGATGTATAAATGTTTGGCTCTGTTTGAGATTTTT

ATCGTTGTCTTTAAAACGAC

**AGO3** (gi|164605505| argonaute 3 [Bombyx mori])

PF: TAATACGACTCACTATAGGGATGGCGGATCCAGCTCGTGG

PR: TAATACGACTCACTATAGGGCGTAAGCTGCTGTATATCCG

ACTGTTTTTTTAGGCAATGTGAATGTTTTTTAATCAAACAGAAAAGAAAATATGATGGGT

GGGGTAGTCATAAATTTTATTGTACGGGGGGTTAAGTTGTTAGTGATTCAAATTCATAAT

ATCATTCTGTTTTGTATAATTGTAAGTTTGTGTTGAATTAAAATTTCAGTGCGTACAATT

TTCTTAACGTCTATGGTACCATTCTCTCACTAAAAACTTGGTAACTCTATGTACAAGTTT

GTTCTTGTTGTTGTTCGTCTGGTATAGCGTGTTTAGTTCAATAATATAGTTATTATCATC

AATATTTTGATAATTTTGGATAATAGTGAAATAATATAGAGTGTGAATATGGCGGATCCA

GCTCGTGGTCGGGGTAGGGGTTTAGCCCTTCTTCAAGCTCTGAAGAAAACTCAGATGATA

GATTCTGAATCATCATCATCATCGGAGAGTCCAACAATGGCGAGCACCCCAGAATCAAGT

ACTGCTGCACCTAGTGTAGCTCCTACCTTAGAAAGTACTACAACATCTATTGGTGGTAGA

GGTCGTCTTGCTGCTATGATGTTAGCCAAAGCGCAACCCAAAGTTGGACCTCCTCTAGCT

CCTGCTTCAGATGTGAGCCCCAATGTACCTCAAGGAAGGGGGGCTGGCAGAGGATTAAAA

CTGCTGCAGACTCTGCAAGCTTCCAAGGCTGGTGAGGTTGCCAAGTCGGATATACAGCAG

CTTACGACTAAAATGGCTGGTAGTTCAATATCAGAAATGCCGCCATCTGAATCAACTCAG

GCGACATCTACTCAAGCAATGGCTACAACCAAATCAAAGAAATATTTTAGGGAAGTGAAG

GAGACTCCACCAGTAGTTCGTAAGGGTGAGAGCGGTAGACCTGTGAATGTGACAGCTAAC

TACATATACCTTAATTTTGAGGAGAACAGCGTCTATGAATACGAAGTCAGATATAGTCCA

GATCAGGATTATAGAAATCTGCGCTTCAAGCTTCTTGGTGAACACAGCAAATATTTTAAA

GAGAAAACATTTGATGGGACTACATTATATGTGCCACGAAAATTACCGGATGAAGCTTTG

AATTTGGTTTCTACTAACCCATTTGACAACACCAAAGTTAATATTACTATCGTGTTTCGT

AGAACACGCCGTCTAAGTGAAATGATACATATATACAACGTTATTTTTAAGTGCATTATG

AAAGACTTACAACTCATTCGATTTGGACGTGATTTCTTCAATGAGAATGCGGCTATACAA

ATACCTCAACACAAACTTGAAGTTTGGCCTGGTTATGTGACAGCAGTTGATGAATACGAA

GGTGGTTTAATGTTGACACTCGACTCAACACATCGCGTGCTCAGAACTCAAACAGTACTG

TCCTTCATCAAAGAGACTGTTCAAGCCGAAGGTGCAAAGTGGAAGCAAGCGCTCACAGAA

CGTCTCGTTGGATGCTCCGTTATGACTACATATAATAAGAAACTGTTTAGGGTAGACAGT

ATTGATGATAGTATGAATCCAAAATCAACATTTGAAAAACTAGAGAATGGTCAACCGGTC

AAAATTACATTCCTTGAATATTTCAAAAAGACATATGGTATCGAGATACGTGATTGGGAA

CAGCCGTTGTTGATATCCAGGGATACGAAGCGCGTATCCGGCTCTGAAAAGCCAGTGGAT

TTTATGATATGTTTGATACCCGAATTATGTCAATTAACTGGATTGACTGACGATCAACGA

AGTAACTTTAGACTTATGAAGGATGTCGCTACATACACTAGAATCACGCCTAATCAGCGT

CATTCGGCGTTTAAAAAGTACATCCAAACAGTTTTGAGTAATGAAATAGCGAAAAGTCGT

CTAGCTGGTTGGGGCTTAAGCATCGCATCAGAGACCGTAGATTTGATGGCGAGAACACTT

CAACCCGAAACTCTATACTTTGGCAACAATGTTAAGGTACCTGGTAAACCGAACGCCGAG

TGGAACAATGAAGTTACAAGGAATGCTGTGATGCAAGCTGTGGATATAGTTAAATGGGTA

TTATTACACACCGATCGGGACAAACAAGTGGCAAGGGATTTTCAAGAAACTCTAAAACGC

TGTTGTAGTCCAATGGGTATTCAAGTAAATAGGCCTGATGTCATCTGCTTGCCAAACGAT

CGTACGGACACATATGTAATGATGCTGAAGAAAATCATAATTTCGGGGGTACAACTCGTC

GTTGCCATCTGTCCAACTGCTAGGGATGACAGATATGGGGCTATTAAGAAAATTTGTTGT

GCTGATAATCCTGTTCCGTCTCAGGTTATAAACGCCCGTACGTTAATGAACCAAGCCAAA

GTACGTGCGATAACACAGAAGATCCTGCTACAGATGAACTGCAAACTTGGTGGTACACTA

TGGCATATCAACATTCCATACAAGTCTGCCATGGTTGTCGGTATTGATGCTCATCATGAT

GCCGCTAGAAAGAAACGAAGTGTTTGTGGATTTGTAGCATCCTATAATCAGCCTATGACA

CACTGGTATTCACGTGCAGTTTTCCAAGAGCGCGGTCAGGAAATAGCAGACAGTCTCAAA

TCGTGCCTTCTGGACGCTCTGAAACATTTCGCGAAAACTAATGCTACATTCCCCGAAAGA

ATTATCATATATAGATACACAAAGATGGCAGGCGATAATAGTGATGGTGAGAAGAAGAAA

AAGAAGGGTAATTGGGAACCTGACAGTATGCATAAAGCCGTGCAAAAGGTGCTGAGCAAT

GAAAGGAGTGCGCGTCGGGCCGCTGCTTTTAATCGAGTCTCTCGATTGGATATTAGCGTT

AATGGCTTTCGTTGCACTAGTATTAACTCCTTTAATAAAAATATTTTCAGTGATTTAGAT

TTTATAGCTTCGAATATGACCAATGTTACAGAAAGTTATCAATCACAGTCAGAATCATTG

CTTCCAAATTATCATCTGTCAGAACCGACCACTTTAGTTTTTGAACAGTCAGAGCTGAGA

AATTTACCGCCATGTCAGCAGCCAGAAATGATACCTCCAGGTCCTCAACAGTCTCACACT

GTCACCTCTCAACAATCCAAGCCTCAAGAAACTACTGTTGAAATTGACCTATCGGTTGAT

AATTTTAAGGCAATAGTTGCGGAATTGTCACCTATTCCTGGTGCTTCAAAGCGACGAGCT

ACTGCCCGACGTTCTTAAGCCGAAAAAAGTGAAATTTACACAAGTTCTCCATAAAAAAGA

TGCTGGAACAGAAAATGACAAAAGTGTTGAGTCAGCTAAGCGAGTTAGGATAATGCTTTC

AGGACCAAATACCAGCAAGAAAGGCAAGAACTTGGTTTCAGGAAAAGATGTCAGCAAGAA

AGGAAAAAAATAAGAAAAAAGAAAACCAATGGCCTGTCCACAAACCAAACGAGGAAAAAA

GGCCATTTGTCCTTTGTGCCTTGAATTTCATGAAGAGGATTGGATTCAATGTGGAAACTT

AAAAGCATGGGTCCACGAGGCGCGTGCAAATATACCAGATCTAACAAATCATTACACACT

TGTGATTATTGTCTAATATAAATGATCATTTTATGCTGGCCGATACTGGAGGCCATATTT

TTTTTAAGTTGATTTTTGTACTCTTCAAATTTTATTAATAAAGTTGATTATTTCTAAATA

TAGCGGTGTTTTATTGTTTTTATTAAGAGAAATTGAATGCCTGAACACATTTTAGCTGAT

ATTTAACATTCAACCTGAGGACTTTTTATTTTATCTTACATAAACCTTAAGGTGACGGTA

CTGCCGGACTCTCCCCTAACACAGTATGCGAAGCAGTGTATATAAATATCGTCGAAACTG

ACAAAATTTTAAATAAACTACATGTAAGCATCGTCACCAGAATCAATAGAATTTCTAATG

GAAATTATGCGTGCAGACCAGCATTATAAATTATAAAATAGTTCTTGAAGACATAGTTTA

GTATCAAGTAATTGCAGCAGTTAATTTATTTTTTTAAAAAGAAATATAATTTAAGTGAAT

ATTAAATAAAACCATTTTTAAGTATTTTATCGCATTTGTATCTTTAACTTGCTCAACGTT

TCGAACATTTTCCAGCGTCAAAATATTTTATTTCATACTAGCTGTACAATGTTTAGGTTT

GAACTTGGAAATAAGATTTCCCTCGTTTTTTAAGACATTTAGCAAAAATGTGTAACACTC

GCGTAAACATCAGAAATCA

**Aubergine** (gi|166706856| aubergine [Bombyx mori])

PF: TAATACGACTCACTATAGGGGATATTCAACAACGCATGGC

PR: TAATACGACTCACTATAGGGCCATAGGATCAGGGTGTAATC

TCTACGGCTCCACCAGTGAGGGCTGGTGTCCCCACACCAACTGTGCAAGTAGGGCGAGCTTCACATCGATCTACACCTACTGTACACCAGGAACATCCCGGCGATGTTGATATTCAACAACGCATGGCAAAAATTGATATTGGACAACCATCTCAGGCCACTGCTGGTGGTGATGCTGGAGCAGTAAGCGGTCGTGGTTCCCGTCGTGGAGGCGGTAGAGTTTTACCTGAACAAATGACTATATTGCGTACTCGTCCAACTACAGCACATACTAAAAAAGGAACCACAGGAACAGTTATAGATTTAACAGCAAACTACTTTACTGTAGAAACCACTCCTCAGTGGCGTTTGTTCCAATATCATGTAGACTTCTCGCCCGAAGAGGACAGTACAGGTGTGCGTAAAGCTCTGCTTAAAGTTCATGCAAATACTTTTGGCGGGTATTTGTTTGATGGGGCAGTTTTATATACTACCAAAAGATTACACCCTGATCCTATGGAACTATATTCCGATCGTACATCTGATGGAGCACGTATGAGAATTATGATTAAGCTGACATGTGACGTTAGCCCGGGCGATTATCATTACATTCAAGTATTCAATATCATTATAAGAAAATGCTTCAATATATTAAAGTTGCAATTGATGGGTCGCGATTATTTTGATCCGGCCGCAAAAATTGATGTACCAGAATTTAAATTGCAAATTTGGCCAGGATACAAGACTACTATTAATCAGTTTGAAGACCGTCTGTTGATGGTGACTGAAATCGCTCATAAAGTACTACGATTGGATACTGTACTCCAAATGCTAAATGAGTATGCTTCTACTAAAGGAAGTCAATATAAAAAGATATTTTTAGAAGATGTAGTGGGTAAAATTGTGATGACTGACTACAACAAGAAAACTTACAGAATTGATGATGTGGCATGGAATGCTTCGCCACGTTCTACTTTTAAGATGCGAGAGGAAAATGTGACCTACATGGATTATTATAGCAAGAAATACAACATCCGCATTCAGGATGCCGGTCAGCCTCTGCTGATTTCGCGATCAAAGCCGCGTGACATCCGTGCCGGAATGCCTGAGCTTGTCTACCTTGTCCCTGAGTTATGTCGTCAGACTGGTCTATCTGATGAAATGCGCGCTAATTTCAAATTAATGCGTGCTCTAGACACACACACCAAAATTGGTCCAGATAGGAGAATACAAAAACTAATTAACTTTAACCAACGTTTCACACAAACTCGTGAAGTTGTTCAGGAATTGGGATCTTGGTCATTGAAGTTGTCGAAGGATTTGATTAGATTTAAAGGACGCCAATTAGCCCCTGAACATATTATTCAAGGCCATAATGCAAAATATCCAGCTGGTGACACCACTGAAGGTTGGACGAGAGAGATGCGGTCGAAGGAATTACTGGCCATTGCTGATTTGCCATCGTGGATTGTAATAACGCCAGAAAGACAACGGCGTGATGCCGAAGGTTTTATGGATTTGATTATGAAGACTGGTTCTGGTGTAGGCTTCCGTATGCCCCGACCAGAAATGGTAACTATAAGAAATGATGGTCCCATGGAATATGCAAATATGTGCGAGCAGGTCATAGCTCGTAAAAATCCAGCATTGATTCTATGTGTTCTCGCAAGAAACTTTCCAGATAGATATGAGGCCATAAAGAAGAAGTGCACTGTAGACCGTGCAGTACCAACACAAGTGGTGTGTGCCCGCAACATGACAAGCAAATCGGCTATGTCCATCGCAACAAAAGTCGCCATTCAAATTAACTGCAAACTCGGCGGTAGTCCTTGGCGAGTGGACATTCCTTTGGCAAGTGTAATGGTGATTGGCTATGACGTATGCCATGATACACGGTCCAAAGAGAAGAGTTTTGGTGCATTTGTGGCCACTATGGATCGACACATGACTGAATACTATTCGGTGGTGAATGCACACACATCTGGAGAAGAACTGAGTGCTCACATGAGTTTCAACATCGCCTCAGCTTTAAGGAAATACAAAGAGAAAAACAATGCATTACCGGGACGAATTTTCATATACCGCGATGGAGTTGGCGATGGACAGATCCCATATGTCCACAGCCATGAGGTTAATGAGATTAAAAAGAAATTGACTGAAGTGTATGCAGGCGCAGAGTACAAATTGGCATTTATAATAGTATCAAAACGTATCAATACACGTATATTCCTGGATCGTGGTCGATCTGGTGAGAATCCACGTCCTGGCACTGTCATTGATGACGTTGTCACATTGCCTGAACGATATGACTTCTATTTGGTTTCGCAAAATGTTCGTGAAGGCACCATCTCGCCCACCTCATACAACATTATCGAAGACACGACAGGCCTGCCTCCAGACAGGATTCAGTGGTTGACGTACAAATTGACTCACTTGTATTTCAACTGCTCTGCTCAAGTTCGTGTGCCATCTGTCTGCCAGTATGCTCATAAACTTGCATTTTTGGCAGCCAACAGCCTCCATAACCAACCGCATTACTCGTTATCTGATACTTTATATTTCTTG

**Dicer-1** (gi|157120736| dicer-1 [Aedes aegypti])

PF: TAATACGACTCACTATAGGGCAGATCCTGGTACCTGAACTG

PR: TAATACGACTCACTATAGGGCAGCATCTTCTTTCTCTTGTAG

TGTAATACTCGCGTAAACATGAGATATCACTATATTTATAGACAAAAGTCTTGGTGACAG

ACTCAAAGACTCTAACTAGGAGAGTCTGCCGCAGACTTGACAAACTCCTGTCAGTGGCCC

CTATGTATCCTGAGGTGACGTAGAGCACATCTAGCGGCGGTGCCTTTCGCGATGCGATAA

TTCCTGCCGACACCCTTGAAGGTGCCACGGCCGAAAACTTCGACGCATACGCGGACTCGA

CGACCATCAGCTAGGCGTTCGGGTTTACCAAACTTGGCAGTGTCAGGTTCAGCTTCTAAC

AACTCTCTTACGGGAGACTTGGGTGCCGCAGCGCTGAAGGCTTCAAGTTCAGCTCCCATT

AGATTCACGTAGGACTTCCAAACGGCAGCCAGCGACATACCTGAATCGAGGAATATAGCA

CCGGCTACTGATTCGAACAAATCACCAAGGGCTTTGGGCACCTCTACATCTTCAGCTTGT

TCCATTTCATCTTCTTGAATGAGGTAATGCTCTTCACTAATAGAATGTCCATTCTCCTCC

TGTATCTTCACATACTTCGTGAGAACTTCGTTCAGGCCCGGAGACATGTGTCTAAAGTAT

TTGTGAAAGCCATGTCGAGCTGCGAGGGTAGCGAAGATGGTGTTATTGACGAGAGCCGAA

CGAAGATCAGTGAGAGCGCCAGGCGAATGCCGTCGAGGATCCTCGTATAAGTGACGGGTG

ATCAGGTAATCCAGTATGGCGTCACCGAGGAATTCCAAACGCTGGTAACAATCTGTCAGA

CGGTTACTGTGATGAGAAGCATGGGTGAGAGCTTGGAGGAGCAGGGAGCGATCCTTGAAG

CGATATTGAAGAGTACGCTCCAGGGCATCGTATCCAGATAGCATTTGTTCCAACTCCCCT

TCGGGGTCTTCGATATATCTGAGCAATGGGGATGGGGGTGCTTTTAACGCACCGAATATT

GGCTGAACCCAGTCACCGTTTTGATCTGCGTAAGGTTTTAATGAGCCGACAGCACGACCA

GGCCACGACCAGTCTGTATCAGGGAACAACTCCTGGTGCTGTTGGGGTTCTTCGTCTGAA

GAGGAGCACTCCGATGACGAGGAGGCGGTACGGTGTCGCTTGGGTGAAGGCGGTTCTGGT

GGTTTACTCTGATGCTTATCAGCGTCCTTAGCTGCCATCACGTATGGATGATCAGTCTCC

AACTTCACATGATGACAAGGTAGAACCTTGATACCAAGCCACGACATGAATAACAAGGCC

CCTCTAGGACCACACTCCAATAGGTAAGCACCTATGAGGGCTTCAACGCAATCCGCTATT

GATTTATCAGGTATGCTGTGCTGAGTGATCAGGTTGTATGGTATGAAGCATCTTGCCGCA

TCCAATGGCGCCTCAGGATTCAAAGCCTCCATTTGAACGTCGCTGTGTAGATTAGGATGT

AGGGTTGGGGGTGGTTTGTAGCAGGGCGGCAACCAATTGTCGTGCGGTTCGAACTTGGAC

GCAATCATTCGTGCACCTAGTCGCTTGTTCCTCCCTAAACGATACAGGTTTAGATTCGAT

ACCTGCTTACTCCTCATGTGACTGAGTTTTCCTTCATGGACAGTCGGATGAGCGCAGTAC

AGATATGCGGTTATCGCGAACTTGAGGAAACTGTCTCCTATAGTCTCCAGACGCTCCAGA

TTGATGCCGTCATTGGCATTCGACATTGTTAGCGCCTGCAATATAACACTAGGACTAGGT

CCAGGATGTCCTTCCAACTTCGGCTGATAATCAAAATCGAATTCAGCTCCACTCTGCTTC

CCCTCCTCATAGTACGGCGTGAACTCCTCCCGTCTCTGGCAAACACCTTCATAACTATCC

TTCCCATCGAAACCCATGTTAACCACCTTCTCATTCACGTATCCTGGCGAATCAATGTCT

ATATCTTTCATAGAGAAGCAGTTGAGACCATCCAATTTTTCTTTTCTTAATGTTTTTTTA

ACACCTTCCAATAAAATTCTTTTATTCCTCTCTATACTGCCAATATCTATTTGACCGTTC

CTGAATGATAGGGCATCAGTGCCGTACGGGAACAACTCCTCAATCGCAGCATCCCACGTC

AGGTCTCCTGTGGGTGGCTGTTTACACTTGCTCTCCGGTAGAATCTCATCCAGGCACTCG

TCTTTGGTGATCAAATTGCCTTTTTCTATTATATCCTTTTTATGGCGGACGACTGAGGCG

CGGAATCTTTCTATATACTCTTCAGGCGCAACGCCTGTTCTCATAACTTTATCATGTTCC

TCTTTATCCAACGCATCGTCTGCTTCGTCAGGCATTGGGGCTGGGTTTGTCTTCTTGGTA

CGTTCTAGATCTACGGCTTCCGCTTCGTGGGCTGTTTTGTATTCTATTCTCACGCCCATA

GCCATATTAGTATTTCTGTTGCAACAAAACCCGTTGTCAGTTTCCTCATCACTCCCAGCG

TAGTCCAAATCAGTATCATCAGAGTCCAGATCACTTGACATATATGAACAGTCACTATCC

ACCATCGAATACATCCTGTTGGGGTGGTACTGTGAAAATTTCGGTTTCTCTATGGGGTCA

CACCAGTTAGTACCACCAGAAGCAGAAGTACAGAAGGTAAGGTTAGGTGGTAACGGTTCC

ATGTATTCATCGTACTCCGAATCCTCTGGTATTTCAGCGGCCATATCGTTACTCCATGTT

CCTATTTCGAAACCACCTTCAGCATCTTCTTTCTCTTGTAGTATATCGTTGATAGTCTTT

TCTCTTATTTCCTCTGGTTCATTCTCCTTTTCCTCTTCCAGCGCCTCCTTTTCCTGTTTA

TCTTTCTCCTGTTTCTCATTTAGCTCCTTCTCTCTTATCTCTTTTTCTTTCTGTTCCTTC

TTCGATTCCACTTCGTCTTTTTTCTTTTCATTCTTCTCCGAGTCCGCACTTAACACCTCG

GCCAAACTCCAACCGAAATCCAAAGCAGGCCACTGGAAACCAGGCATCGTCTCTTCGTTA

ATCTTTGGTATACCAAGTCCCACATCAATGGCGACCGCGCGTCGAATCTCATCGGCTATC

AGCAATGCATTTATTCTGTATAAAACGCATGGCAGCGCCACAGTCGCGAACCATAGTGGA

GCTGCGAACGGATGCACTCGACACAGTTCAGGTACCAGGATCTGTTTCTGATCAAGCCTG

TCACGTTTGGCGCGCCTGGTCCGCTCCGAGGATACTGGAAGTGCTACGCCTTTTCTATTC

ACATATCTTGGTGTAAGTAGATTTAATCTAGCGCTGGTATGATCCACATCGAGCAATGGC

TGTTCCTTCTGCGTGAGGGTCACACCGTACTTGCTGCTGTAGTACTCCCTGAAGCTGGCA

TGACTCGAAGACGGGAAGGAGGAGTCAGGTGTTAAGTTCCAGCAAATCTCAGCGACTAGG

AAGAACTGCGGTTGATCCTGGTTCCTATACCAGGGTGTGACTACGGCCTCCTTGTATTTC

TCAGCGTCAAACATGAAGATCTCGTCCGGTTTCAGCAGGGGATTGTCCATCTTCTTTACT

TTGTTCTTTTTATCGCGTGGATTTCTCGTCTTATTCTGCTCATCATCTTCTTTATTATCC

CATAATATTGCGTTGTCGACTCCAGATATTTTCTTGACTTCTGTATGCTGGTAGATTAGC

TCGAGGAATGACCAGTCAATGTCGATCTTCGTGGTACCATCCGGTCGAATCGATTTAATT

GTGGGAACAATATAGTAGTTATTGTGTGTTGATCCCTCGCTCTGTAACCTCATGCCTCGA

CGTCGAACCCGCAACACCTCGGCAAACACGAACCGCATGAATCTTCTAATCAAGCGTCCT

CTTCTAGGTGATAATCTGACATCAGCGTCTGGTACATGTTCCACCGAGACCCTTACCTCT

CCTGATCTCGTGTATACAGGAAAGGCTGGTATCTGCAAACTATCAGGTTCCTCTCTAGCC

ATAAGGATGCCTATGGCCTGACTGGCGTTCTGTGGTGCATGCAATCGCCTCCCCCTAGTG

TTATAACGTTCAGGTAGCGCACACCATAGCGATGAAACAACGGCATAGAGCATGTTCCGT

TTTGGTCCTGTAGGTTTTTCTTTTGGAAGCTCAGCTTCGGTGTCATCCTTTATCTGTTCC

AGGGAGTCTACCTCACAATCCCTGGTATCCACAATCGGTTGGCAGTCCGTGAATGCCCAG

GCAGTCCTTTTGTAATAATACTGTCGTCGTTTCGTTGTTCCTGGACGGGCACTATCGCTA

TTATCAATCGCATCATTATTGGTACAGGCGACAGCATTTGACGTTTCCAATTCAGCCGCT

TTGAAATTCTCTTTACCTATTGGCATCAGCTGATCATCCAATTCCCCAGACTTATGCAGT

ATCCTGCACGCCTGCAAAGCTACCATCCTTCTTGCTAAAACCTTCGTTGGCATTGGATGA

CCCACAATATTATATTTGACTGGACAATTCAATGGTAGTCTCAAAGTGCATATGTATGCT

GTTCTCATCTCCTTGGTGTCTTTATGTGGAAGCTGTACTGTCTCCATCCACCATTGAGGT

GCCAGGCGGGTGAAGGTATCTGATGGTAACTTGCCACAGTACCTATTAATAAGTGCAATT

GCTGTATTCAAATCCACGCTGGCAATATCATTATCATTTAATATTTTTCTATTATCGCTT

TTGCTAATATCATTATTAATATTATTATCCTTTGGCACACAAGGACACACATCACTCTTC

AAATCACCGCATTTATTACAAAAAACATTTTTATCCCTATCACTAATTTCATTTACAATT

TCATTTATATCCTTTTCTATTGTTTCTGGAATGTTATTGTCTATGGAATTATCAATATTA

ATTTTTCTATTATCATTTTCACTAATATCACTATTTTGTATATTTAGATCGTTTGTGACA

TGTTTTTCATTGTCATTAATATTATCGTTTTTATTATTCATTTCAAAATCACCGCAAATT

TCCCAATCGTCTGTATCTTTAAGACCATCTTCGATTTTTATTTCGGCTGTTTTATTTGTA

ATATCATCAATATTCTTCTTATTCTTTTCACTAATTTCAGTATTGTGACTACCATTAATG

CTCCTATGACCATCCAAGAACTTATCATGATCTACATGATTATTAGTTTCAGTATTTTGA

CTAGCCTCTAACGCATCACCAATGCTTTGATCAGTATTAAATTTACTTCTAGCCTCGTTT

TCATGACTATCACTATGACTATTACAATCATTGTCATTTTTGTTTGAGATCTCACTGCTA

TCATTATTTAAATTCGTATTGTCTTTTTTGCTTATATCATTATCAATATCTATAATGCCT

TTATGTAAATTAGCTCCAATAAATCGATTATCATTCTCGCTAATAGTTTCTACTTCGCTA

ATTTTATTAGTAATTTGTTCATTGATACTAGTGTTATTAAAACTATCATTTGGTGTATAA

GGTTTAATAAACGTGTTTAGTCTATCAGCATGATTTTCTTCTGCTGGAGGCGGTTCATCT

TGTATGCCACAGCCACATTTACGGCTTATTATCTGATCCAATTCCCTGTAAGTGGCTACA

TTATGAAGCAAACCCTCGGTAGTTTCAACAGAATGACTGCAAAGTAACGCAGCGGTTGCT

CTCGAAGCCCTGGCTCTACCACGGCAGAGTCCATACGAACGGTACGAAGGTGGAACATCC

CAACGTATCACCAGGTTACATCTAGGCAGATCGATACCTTCCTCTAAAGCTGATGTAGCC

AACAGAAGATTACATTCGTGCATACGGAATTTTTTAAGCACTTCCTCCTGTTTCTTTCCT

TGCCGCTGACATTCCTTTAGCTCAGTGCCAGGGTTAGCTGGTGTCTCCGTGGCACAGTAC

TGAGCGCATAGATAAGACAGGCGAGGGTTGCAACGTGACATGTCCACTATTATCATGAAC

ATAATTTTCGCAACCAAAGCATCTTTCATATAGACAATACCGCATAACGCATCTGGATTC

TGTTGCATTTGAAGAACCCTTGCAGCATTATTTCTTTGCACCCGAAGTCTTCCACGAGTC

CTATGCCCTGTACGCCTGGGCAAAAGAATTAAACCCTTTGGAAACACCTCCTGAACTTTA

TTTTCTGATATATCACTCTTGCTAATATCTTTAATTGTATCTATCTTTTCTTTATTAGCT

GTAATATCACTTACACTAATATCCGTATCGTTAATAGCTGCTAAATTAGCGGCATTATTG

TTGTTAATATTACTTTTAGAGGTATTATTCTGGTATTCATTAGAATAATCCTCAATTTCT

TTTAAATTAGCTTCGAAAATATTAACTTTATCCTGTATTCTGTTGCCTAAAGTTTTAAAA

TCACATTTATTTAAATCTTTAAGCATTTTCTTAGTATTATCTTCTCTGCTAATTTTTGTA

TCATTCCCGCTAATATTTGTCTCGCTAATATCCTTATTGCTAACTTTTGTGTCTGGTGGT

TGAAAAGCTTCTAATATTTCAGCGAATCTTAATACTTTTGGGGCGCTGAAAGTTTTGATT

TTCTGCCATTCCTTATCAAACTTATTAAACATATAGTCTGCATAGCCTCTGATTTTAACA

AATACCGTTGTGCATAGACACAGTAATAGAAAATGTCTTTCATATGGAATTTTCACTTTT

AATTTTTCTAATTTAGTTAATAAAGCAAACGCTGCTTTGTCCGCTGCGTAGGGTCCAAAC

TGTTGTAGTACATACAAGAACTGCTTGAAGACAAGTTTAGGATCCACCCCTGGATCCGGC

ATGTTCATGAACTCCTCGTACAGCTCATCACCATATATCTCAGTGGGATCATGCCTATGG

TCTTCGATAAATGCCATGGCCTCATTGATGGTATCCAGCATGAAACTCTCCAACTCCTTG

TATTCCTCAGATATGGTGTCATCAGAAGCACGGAAACCATATTCGATTATCAATTCCCTA

GGTTTAGCAAGAGAGTTTGATATTCTTTTCCCTCCATCAATATCCTCGGCCAAGTCCATT

TGACAGCATAACTCATTCTCCAACTGTTCTATCTTCCACTCCAGCTTCTCATATAAACCA

AAGTCATCCAAGTTCTCATAAACGCCCAACTCTTCTTCGTTACCCTTATCTTTCATCATC

TCCTCTTGGCTCTTGATACTGATGATCTCTTGCGGGTTCTCAATCACTGGTATCTCTTTC

TTCGTCTCTTTCTTAGGTGATGCAAACAGTGGGTAGGTCATCGCCAGAATGATGGGACGT

TTAGACAACGAGCAGTGTTTGTATGCTTCCATTACATATTTAAGTTGGTCATCTTTGTAT

ATTAAATGGCAACTGTCTACCACCAATACGTTGATGGCAGTTATCGTCAGTAGCTCCTCC

ATCAATAACTGCTGCAGTACCGCCGCTGTGCATATTATAACCTCTTTCTCCTCCAATTCA

GTATTCCAATCGACCACAGATCCATAAGTTTCAGCACATAATGTGGCAAGATCAGTTAAT

ATCTTCAACTCGTAAGCAATCACCTGGACCTTTGTAGCCCTTGTCACATATACAGACCTC

TGCTTACCTCCAGATAAAGCTCCTCTTATCTGATAGGCCTTTTCATACAACAATCGTACA

GGTATGAAAGAAGCATATTCCACAACAAGTATATTCTTAGTCGCTGCAGCAGCTGCCAAC

CGTGTACCACCTTCAGCTGCAGTAAAACGGGAACAGCAGGCTTCCATTATGATCACGTGT

CACAATAGTCGTAACAAAGTAAAAATATTATGTTAGCACATTATTTCTTAAGGAACAAGA

ACTCAAACAATCGTTAACACATCGAAATAAGTTTTCAAAACTATAGTGTTATTCGATCTT

AATAACACTTGATATTGGACTTATTACAAAACAAATTGCACTTAAATTG

**Dicer-2** (gi|302318908| dicer-2 [Bombyx mori])

PF: TAATACGACTCACTATAGGGCCAAGTTATTGTGATGACGAGTC

PR: TAATACGACTCACTATAGGGCCAACAATAGTATCAATTAAC

TCGCGGCCATACCAAGCCCAACTTGAAGAAATTGCAATAAAAGGCAACACTATCATATATTTGCCTACAGGTTCTGGTAAGACATTCATTGCCATATGTCTAATTAAGAGATTTCGTGAAGCTTTACAAAAACCATGGGGAGAAGGCGGCAAGCGGACGTTTTTCCTTGTAAACACTATACCGCTCGTCACTCAACAGAAAAAATGTATTCAAAATCTATGCGCCATTAGTGGTGTAGGTGGGTATAGTGGTGAGGATGGCGTAGATTATTGGGATAAGGAAAAATGGGATGCTGAGCTTCAAGAAAACCAAGTTATTGTGATGACGAGTCAAATTTTATGCGACATGTTGACCCATCAGTATATAAAGATTGAAGATATTAATCTTTTGATATTTGACGAGTGCCACCATGCTGTTGAAGATCATCCTATGCGGATTATAATGAAACATTTTGAAGACTGCCCCAAACAAAAACAACCAAGAGTTCTAGGGTTGACAGCAACTTTACTGAATGCGAACGTGAAGACATCCAAGGTTGAGGAGATACTGCACGATTTAGAGATCACCTTCCATGCGACTATCGCAACAGTAGATGAGCTTGGCAAAGTCTTAGACTACTCAACCAATCCCATCGAAATGGTTCAAACTTACAGACAATTTGAACCGTCCGAGGCTTCTAAAAAAGTCATTGCTTCATTGACACAGTTAATTGATACTATTGTTGGTACAAAATTACCCCCAAGTCGGAACATTCACAATGTCAAATTAAAACCCGGCCAAAAAGACATAAGTACTGATCCTGCTAAGGTGGTGAAAGCTGTGAAAAATATGGTAGTAGCGATGATATCGTTTATTCAAGAGCTTGGCGCTTTTGGTGGTCTTGTGGGAATAATTGCTTATAGAATATTATTAGAACGCCTAAAGAGAACAGCGTCTACGAAACAAGAAGAAGTACTATATAGAACTGTTATCAACCACACTGTCGTGGCCCAAAAATGTTTGGAAATTTCAATGAAAAATGAAACTGGATATGATAGGATTGTTAAACATTCATCAGAACAAGTACTGTTACTTTTGAACATATTGAAAGAATATAGTCCAGCATTCATGAACAAACCGGGCGTTTTATTGAAAGTGAATCAATCACGGAAACCGCTATCAGCGATAATTTTCACTAAACAAAGGTTTACAGCAAAAGTGTTATTTAACATTTTAAAAGCAGTGCGAGAGGCAAATCCTGCAGAATTCGACTTTTTAAAACACGATTTTGTAGTCGGCTTCAATGTAAACCCAACTAAAAGTACCAGAGAAGATCATTATTCTAAGAAATACAGTCAGCAAGCACTTTTAAAATTTAAAAATAACGATCTCAACTGCTTAATATCAACAAGTGTAATAGAAGAAGGGATTGATATACCGCAGTGTCTGTTGGTGTTACGATACGATCCGCCATTGGAATATCGCTCATATATACAAAGCAAAGGACGTGCACGTAGTGCCGAATCCAGTTTCGTTATATTGGTCAACGAGTCGGCAGAAAAGAAATTCATGAATAATTACCACGAATTTCAAAACACCGAACGGTTAATACAAAGAATATTAGTTGGTAACAGTGAAGACCGAAATGCACCTGCCACCAATGATATTCATAATCAGCTTTATAATGAAGGGGATATAGAGGATTTTGTTGCCCCAAGCGGAGCTCGCTTGACTTGTGTATCAGCTATAAGCTTGCTGAATCGCTATTGTTCAGTATTGCCTACCGATCAATTTACCGTTATCACACCAATGTGGATCCAAGAGCATGTCATGGTTAAAGGCGTGAAACACAGTCAAATTAGCATTTTAATGCCAATTGCTAGTCCTGTGAAAGAAGAGATAAAGGGCATGTGCTTGTCTAATGTGAAGTCTGCTAAACGTTCCGCAGCATTAAACGCTTGCATCAAATTATATGAAGCTGGGGAATTAGATTCTGGATTACTGCCAGTACGATATACATGTGTAGATTTCGATGTTTCTGAAGTAAAGGATATTTTTCCAAGTTGGCGCGATAATGACACTGGACGTGATGGTGAAGATGTTCCAAAGCCCGGTACTAAAAAGAGAATACGTAAACATCCGATTGAGTTTCCGTCTTATCTGGACAGTGTTCCCAATAGTAATACATATTATCTGCATATTATCAAATTGAATCCCGCTTTCGTAGAGCCCAAAGATTCTCGTGAAAGATCCTTGTACAAATTAATTCAAAGGAAAGAAGGATTCGGATTTTTAACTCAGAAACCTTTGCCCCAATTATGCAATTTCCCTATGTTTATGACAGTGGGAGAGGTATCAACTTGCATAGAAGTCAATCATGCCGTTATAACGCTTAATTCGGAATTGTTCGAACTGGTGAAACACTTCCACTATTTCCTTTTCGACCAAGTCCTGGGCATAGCTAAAAGGTTCGTGGTGTTTGAAGGCAAAGTGAATTGCATGTATGTCGTGCCCATAAAATATAGTAATGGATATGATATAGATTGGAATGTTATGACGACCCATACAAGTATACAGCCAGTCCAACCAACATCGTACGAGGAGCGTATATCTGTAGAAGTTACATCGGAGAACTATAAAGATTGCGTTGTTACACCTTGGTATAGGACTTTACCGGATAGATACATTGTCTCAGGTGTTTTAGAGCATATGACGATAAATTCTACATTGGATTCAAACTCTTGCATATCCTTTAACGACTATTACGCAGACAAATATAAATTAGAAGTTATTGGTAACAGACATCAGCCGTTGTTAGAGGTCAGAAATATAAGCTCTAGAATGAACTGTCTATTGCCTAGAGCAGCGACGATTAAAACGTTTACAGACAAACAAAAGAAGCTAATATCTGCTTCACAAGGCGATGATAAGAGCAAAGCCTTCACTGAAGTTTTCGTTGCAGAATTTTGTATTAAGTACGATTTCCCCGGCGTGCTGTGGTACAAGGCCACTATGTTACCTAGCATTTTACACAGGGTACATATGCTACTCACAGCTCATGAACTACTAACGCAAATATCAGAGGGTTTAAATTTTGGACCACAGTGTCGCACTGGCAAAGATAGATACTCAGCTACGGTTAAAAGAAGCATAGAAGAGGAGGAAGAACAAATTACAAAAATGAAAAAGGTTGAAAATGGAGAAGGCCTGAGATTAAAACACAGAAATACTGTAAAAGATGACAAATGGCTTCCAATAACGATAGATATGCAAGTTGCCATTCAATCGTTACTGTCACAAGTCGAAGAACCAACACAAAACAATTCTGTCGATAGAATTAACAATCCATTAGATGACGGCAAGAAACGGCCGAAAATAATGTCTATGAAGGAAAGCTTGTATCAATTACAGCAGAAGAGAATTGATAAAGAATATCCGTGGGATGTAAGAACAGAACCAGTGGACATAGAGCGAAATATTTTAAGCGTAACTGTTATGGACGTGGAATGTTACGACAAGTTTATGTCAGCGCCTTTGATTGATGTGAAGTCAACAAGTGCAGTGCTTTCACCACCACGAGTTATAACGTCGGCGGCGATATCGGCTGCACCTGCCAAGTATTGCGATAAAATTGACCTCTTGCATATCGGTCTTAGTAAGAAAGGTCCCGAACTGAGAGACATGCTGGGAGCCTTAACAACGATTAAATCTAATGATACTTTTGATTTGGAGAGAGTGGAAACACTTGGTGATTCTTTCCTCAAGTTTGCTGCAAGTTTATACCTGTACCATAAATTTCCGAAATTCAATGAGGGTCGGCTTACAAATATAAAGGGTCGTCTTATTAGTAACAGAAATCTCTATTATGCTGCTGAAAAAATCAACTTAGGAGGACGAATGAAGATCGAACAGTTCTCACCAAAAAAAGATTTCTTAGTGCCTGGTTTCTTTGCACCAAAAGAGGTTAAACAGTTTCTGGCAGATAAAAAGATCCGTCCGACGTTTATGATCAGCATGCAGTTTCCGATGACGGAGGTGCTTAATGGACGATTATCTTCAGAAAGTATGGGCTTAGTTAGACAGCAATATGAGATCGAAGGCGGCTTCGAGACAGAACCAAATGGTCGTGCACAGAATGCCATGCAATGTTACGTACATTCACAAGCTGTGGCCGATAAGACTGTAGCAGATGGCGTGGAAGCACTTATCGGTACATATCTCTTGAGCAGTGGCATCTTAGGGGCGGTCAAATTGATTGAATGGATGGAGATAATACCACCGCAGGATTCCTTCGCTGATATGCTTCATAAAAAAGTATCAACGGTTATCACAGATGGAAAAGCAACAACTATGGATATAGACTTTTTGCTGTCACATAGTCGCAAAGACGTTGAAAAAATACTAAAGTATAATTTCAAGGATCCGTCCCTTATGCTTGAGGCGTTATCACATTCTTCGTACATCCGAAACAGGCTGACGCGTTCTTATGAACGTCTAGAGTTCCTCGGTGATGCGATCTTGGATTTCCTTATCACATCGCATATATTTGAAAACTGTCGTGATTTGAAGCCGGGCGAAATAACAGATCTACGGTCTGCTCTTGTTAACAACGTCACATTCGCTGCTTATGTTGTCAAATTAGAACTTCACAAATTTATTTGTTGTGAATTAAACGTCCCGCTATCAAAAGCTATCGTTATGTTTGTTGAACATCAAAATCAACGGAAACACGAAATTGAGGAAGATGTTCTATATTTAATAGACGAAGAAGAATGTCACATAGCTGAATATGTTGAAGTGCCAAAGGTACTCAGTGATATTTTCGAAGCACTAATCGGTGCTATATATTTAGACAGCGGTGGCGACCTGCAAATTGTCTGGTCCGTTATCTACAGGATAATGTGGAAGGAGATTCATTCGTTTGCCGCGCGCATACCGCAGCAGCCGGTGAAAATACTCTATGAGAAGATACATGCGTGCCCAGAATTTGGGCCAGCAGAGATTATGGACCCCGATCTTCCTAAAATAAAAGTTTCTGTAACGATCACTAAAAATGGTCAGCAGTGTACCGTATACGGCATAGGCAAGAACAAGTCGCAAGTTAAAAGG

**Polyubiquitin** (gi|104303708| ubiquitin [Pelophylax nigromaculatus])

PF: TAATACGACTCACTATAGGGACACTGACCGGCAAAACAATC

PR: TAATACGACTCACTATAGGGATACCACCGCGTAGACGTAATAC

GCGCGGTGGTATGCAAATCTTTGTAAAGACACTGACTGGTAAAACTATCACTTTGGAAGTTGAACCTTCTGATACCATCGAAAATGTCAAAGCTAAAATTCAAGATAAGGAAGGCATTCCACCAGACCAACAAAGATTGATCTTTGCCGGTAAACAACTTGAAGATGGCCGCACATTATCAGATTATAATATTCAGAAAGAATCCACTCTGCATTTGGTATTACGTCTTCGTGGTGGCATGCAGATCTTCGTAAAGACACTGACCGGCAAAACAATCACACTGGAAGTTGAGGCCTCCGATACTATCGAAAATGTAAAAGCCAAGATTCAGGATAAGGAAGGCATTCCTCCAGATCAACAGAGATTAATCTTCGCTGGGAAGCAATTAGAAGATGGCCGTACTCTATCAGACTACAATATTCAGAAGGAATCCACTTTGCACTTGGTATTACGTCTACGCGGTGGTATGCAAATTTTTGTTAAGACCTTAACTGGCAAAACCATCACATTAGAGGTTGAGCCTTCAGACACAATTGAAAACGTTAAGGCTAAGATTCAGGACAAAGAAGGCATTCCTCCAGA

**Ub-RPL40** (gi|315115459| ribosomal protein L40 [Euphydryas aurinia])

PF: TAATACGACTCACTATAGGGCAGAGAAGCTCTACAATATG

PR: TAATACGACTCACTATAGGGGAATTTAAATCCTTCAACTTC

AAGCAGTGGTATCAACGCAGAGTACGGGGATTGACAGAACTGATCTTTTACTTTGAGCAC
GTTTTTCAGAGAAGCTCTACAATATGCAGATCTTCGTAAAAACCCTGACGGGTAAAACCATCACCCTAGAGGTGGAAGCTTCGGATACTATTGAGAATGTGAAAGCCAAAATTCAAGATAAAGAGGGTATCCCACCAGATCAGCAGCGTCTCATCTTCGCCGGTAAACAGTTGGAAGATGGCCGCACTCTTTCTGATTACAATATCCAGAAGGAATCCACACTTCACTTGGTACTTAGGCTTAGAGGAGGTACCATTGAGCCATCTCTCCGCATTCTGGCAATGAAGTACAACTGTGAAAAAATGATTTGCCGCAAATGTTACGCCCGCCTTCATCCTCGTGCAACCAACTGCCGCAAAACCAAATGTGGTCACACCAACAACCTCCGCCCCAAAAAGAAGTTGAAGGATTAAATTCATTATATTTGCATTTACATTAATGTAACACAAATATCTTGTGATTAATTACTAAGCCCACATTAATAAAAGTGTAACAACAATAAAAAAAAGAAAAAAAAACAAAAGTACTCTGCGTTGATACCACTGCTTT

**Ub-S27A** (gi|302393750| Ubiquitin-40S ribosomal protein S27a [Manduca sexta])

PF: TAATACGACTCACTATAGGGATGCAGATTTTCGTTAAAACC

PR: TAATACGACTCACTATAGGGTTTGTCATCGTCTTTGAAGACC

ATGCAGATTTTCGTTAAAACCCTAACGGGTAAGACCATCACTCTTGAGGTCGAGCCCTCGGACACCATCGAAAATGTGAAAGCCAAAATCCAAGATAAGGAGGGAATCCCCCCTGATCAGCAGAGATTGATCTTCGCTGGCAAACAGCTAGAAGATGGTCGCACCCTGTCAGATTACAACATCCAGAAGGAGTCCACATTGCACCTGGTGCTGCGCCTGCGTGGTGGTGCCAAGAAACGCAAGAAGAAGAATTACTCCACCCCCAAGAAGATTAAGCACAAGAAGAAGAAGGTCAAGCTAGCCGTGCTCAGGTTTTACAAGGTGGACGAGAATGGCAAGATCCATCGTTTGAGACGTGAATGTACCGGCGAGCAGTGCGGCGCCGGCGTGTTCATGGCCGTCATGGAGGACCGTCACTACTGCGGCAAGTGTCACAGCACAATGGTCTTCAAAGACGATGACAAA
